# Optimization of urolithiasis treatment and diagnosis in the Turkestan region

**DOI:** 10.25122/jml-2021-0107

**Published:** 2022-03

**Authors:** Reza Fathi

**Affiliations:** 1. Medical Center ARAD-RI-ECO, LLP ARAD-RI, Kyzylorda, Republic of Kazakhstan

**Keywords:** urological pathology, risk factors, population health, kidney stones, prevention

## Abstract

The article aims to identify the main problems in treating urological pathologies by analyzing scientific literature from this field and developing recommendations. The quantitative excretion of uric acid, urine volume, and pH are essential in the formation of uric acid stones. The most important risk factor for uric acid nephrolithiasis is the acidic reaction of urine, which is a prerequisite for the formation of urinary stones. When urine is alkalized, the pH should be 6–6.5. Drugs alkalize urine, and one should titrate using urine pH indicator paper until the level is stable. This study found that the spread of genitourinary diseases is increasing. This situation can be improved by monitoring and assessing epidemiological processes, preventing urological pathology, and optimizing medical care organization in the context of health care reform.

## INTRODUCTION

Urolithiasis is a widespread pathology with a high relapse rate [1]. Drug therapy aims to reduce severe pain syndrome, optimize stones passing, dissolve calculi from uric acid and cystine, and prevent relapses [2, 3]. Prescribing non-steroidal anti-inflammatory drugs is prioritized over opioids for relieving renal colic (does not induce vomiting) [4]. According to the results of randomized studies, the effectiveness of anticholinergics has not been proven [5]. With α-blockers, particularly tamsulosin, there is a decrease in pain and an improvement in the passing of stones and their fragments after extracorporeal shock wave and ureterorenoscopy lithotripsy [6]. The use of potassium citrate promotes the dissolution of uric acid and cystine stones and prevents the formation of new ones, even after extracorporeal shock wave lithotripsy or percutaneous nephrolithotomy [7]. Other measures to prevent relapses of nephrolithiasis include a drinking regimen and diet, which counteracts underlying metabolic disturbances, together with appropriate drugs, herbal medicines, and probiotics [8]. After establishing the role of nanobacteria in the genesis of stone formation, the appointment of antibacterial therapy opens up a new perspective for the prevention of urolithiasis [9]. According to various researchers, the range of incidence of genitourinary diseases is large in Eastern Europe [10].

Improving population health and reducing the global burden of disease requires special attention to the leading causes, including the genitourinary system. An analysis of scientific publications indicates a significant prevalence of this pathology among the population and its negative consequences on health and quality of life [2–6]. According to the World Health Organization (WHO), genitourinary diseases affect people of any age, including young people under 40 [5]. These include abnormal development, traumatic injuries, neoplasms of the urinary and male genital tract, infectious and inflammatory diseases, urolithiasis, hydronephrosis, acute and chronic renal failure etc. Moreover, these diseases are the most frequent causes of temporary and permanent disability of a significant population, significantly contributing to the mortality rates and the global burden of disease.

Significant morbidity and prevalence of genitourinary diseases require improved monitoring and assessment of epidemiological processes, prevention, timely and complete detection of urological pathology, and high-quality treatment [11–16]. This raises relevant tasks to optimize the organization of medical care for urological pathology in the context of healthcare reform [17]. Given the frequency of genitourinary diseases and their complications, the scale of the economic losses for society, countries, communities, and individual families is understandable [18]. Differences in the incidence of genitourinary diseases in different territories are associated with inadequate prevention, insufficient staffing of primary care specialists, qualifications, quality of diagnostics, and medical and technical equipment of healthcare institutions [19–25].

The article aims to identify the main problems in treating urological pathologies by analyzing the scientific literature from this field and developing recommendations.

### Features of the long-term treatment of nephrolithiasis using potassium citrate

This study was conducted to examine the effectiveness of selective therapy following guidelines for preventing relapses of calcium oxalate nephrolithiasis and assessing risk factors for relapsing calculus. In a prospective study that lasted two years, 134 patients with relapsing calcium oxalate nephrolithiasis underwent regular examinations at least every six months during this period. Participants were given diet and medication recommendations based on 24-hour urinalysis, metabolic status, and dietary patterns. During the observation period, cases of relapse were recorded in 57 (43%) patients. In individuals without relapses, a significant increase in urine volume, as well as an increase in urine pH and excretion of potassium and citrate (three indicators of the effectiveness of alkalinization), contributed to a significant decrease in the risk of calcium oxalate stone formation [26, 27]. In patients with relapses during the observation period, there was a significant increase in the relative supersaturation of calcium oxalate, mainly due to a significant increase in urinary oxalate excretion. Multiple logistic regression analysis showed that extracorporeal shock wave lithotripsy and a history of frequent stone formation are independent risk factors for relapsing nephrolithiasis.

The urological health of males is determined by the influence of a complex range of medical-organizational, socioeconomic, and demographic factors [28–30]. Based on the study of Tusupkaliev *et al.*, a three-level organizational system for the provision of specialized care for men and young men were proposed, which allows increasing the availability of high-tech medical care and a network of urgent diagnostic urological departments, increasing the detection of all types of andrological pathology [31].

**Figure 1.**
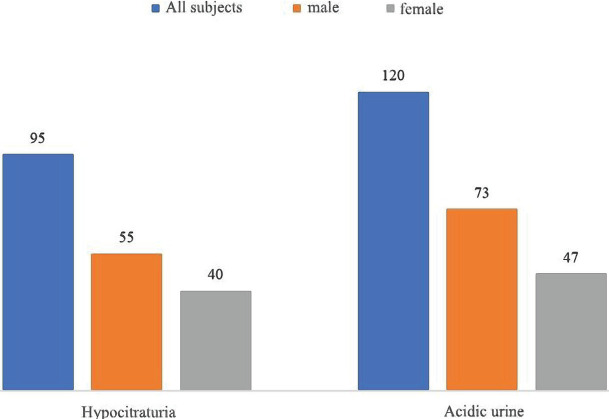
Retrospective analysis of the medical records in the Turkestan region [34].

In the study of Galvanetto *et al.*, patients revealed a tendency towards a decrease in the quality of life in all components, including the physical, mental, social, spiritual, and general perception of their state [32]. The quality of life of patients with genitourinary pathology indicates the need to improve preventive work and increase diagnostic and treatment process efficiency [33]. Turganbekova *et al.* aimed to examine metabolic changes in urine during alkaline therapy with potassium citrate in patients with hypocitraturia of any genesis or excessively acidic urine pH and determine the degree of remission in those patients whose treatment lasted more than 24 months [34]. The authors retrospectively studied the medical histories of 215 adult patients with relapsed nephrolithiasis who had such risk factors for calculus as hypocitraturia (n=95) or excessively acidic urine (n=120). All patients received potassium citrate treatment for more than 3 months (Figure 1).

In patients with hypocitraturia (55 men and 40 women, average age 43±14 years), during therapy with potassium citrate (the average daily dose was 48±14.7 mmol), there was a persistent increase in urinary citrate to normal levels, potassium concentration and urine pH and serum potassium. In patients with excessively acidic urine pH (73 men and 47 women, average age 48.7±12 years), who took potassium citrate with an average daily dose of 42.8±15.5 mmol, a significant increase in urine pH, potassium levels, and urinary acid was recorded. The degree of remission was studied in 35 out of the total number of patients; the average observation period was 31.6±14.3 months. All these patients received potassium citrate in an average daily dose of 45.4±15.2 mmol. As a result, in 91%, stone formation relapse was not recorded. Thus, treatment with potassium citrate corrects metabolic disorders in patients with hypocitraturia and excessively acidic urine pH and prevents relapses of nephrolithiasis, ensuring a high degree of remission of the disease. There are several pathogenetic factors in the development of uric acid nephrolithiasis: acidic urine, decreased urine volume, and hyperuricosuria. However, the most important is the acidic pH of urine, which is a prerequisite for the formation and growth of uric acid stones. Urinary alkalinization with alkaline mixtures is widely used by specialists to dissolve kidney stones [35, 36]. Dobrovanov *et al.* aimed to evaluate the clinical efficacy of potassium citrate/potassium bicarbonate therapy in dissolving radio parent stones [37]. The trial included eight patients with radio parent stones (≤15 mm) in functioning kidneys (four men and four women, average age 66±2 years). Patients underwent ultrasound or computed tomography to confirm the presence of calculi and radiographic examination to exclude calcified stones. In addition, blood samples were taken to determine the level of glucose, creatinine, and uric acid; a 24-hour urinalysis was performed to assess the amount of daily excretion of uric acid. A urine culture was performed to exclude urinary tract infections. All patients filled out diaries at the beginning and weekly during the study, in which data on the volume and pH of urine were recorded.

Three urine samples were collected daily to determine pH and volume (in the morning from 8 to 14 hours, in the afternoon from 14 to 20 hours, at night from 20 to 8 hours). Two study periods were examined: during the first 6 weeks, patients consumed 1500 ml of water daily, while in the next 6 weeks, in addition to the drinking regimen, they were prescribed 40 mmol of potassium citrate and 20 mmol of potassium bicarbonate (divided into two doses). The preference was given to alkaline solutions with potassium to reduce the risk of precipitation of calcium salts [38–44]. The efficacy of stone dissolution treatment was assessed by ultrasound after each study period (6 and 12 weeks). During the first treatment period, the condition remained unchanged in all patients. In contrast, after 6 weeks, while taking potassium citrate/potassium bicarbonate, complete dissolution of stones was noted in three patients. In the remaining five cases, partial dissolution was observed; in two of them, complete dissolution of calculi was achieved after continuing treatment for 4 and 6 months, respectively. The average urine volume was unchanged over the two study periods. The average urine pH was significantly higher during therapy with potassium citrate/potassium bicarbonate compared with the first study period (in the morning 6.60±1.06 *vs.* 5.53±0.51, p=0.030; in the afternoon 6.53±0.70 *vs.* 5.63±0.41, p=0.007; at night 6.57±0.51 *vs.* 5.98±0.80, p=0.092). The drug was well-tolerated, and no serious side effects were observed to interrupt treatment. None of the patients needed additional therapy for urolithiasis. Alkalinisation of urine using potassium citrate/potassium bicarbonate is well tolerated by patients and is a highly effective treatment for non-obstructive uric acid nephrolithiasis.

### Efficacy of the potassium citrate therapy in patients with residual stones

**Figure 2.**
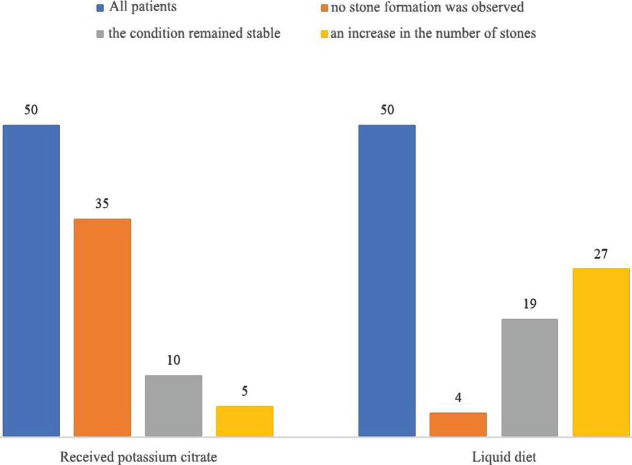
The results of the extracorporeal shock wave lithotripsy therapy [45].

Given the effect on the solubility of calculi-forming salts in the urine, potassium citrate is conventionally used to facilitate the passage of stone fragments after lithotripsy. The objective of Zhumalina *et al.* study was to examine the practice mentioned above and the effectiveness of long-term treatment with potassium citrate to prevent relapse of urolithiasis in patients undergoing extracorporeal shock wave lithotripsy [45]. A prospective study was conducted in the Turkestan region with the participation of 100 patients with calcium oxalate and calcium phosphate nephrolithiasis who underwent extracorporeal shock wave lithotripsy therapy. Participants were divided into four groups of 25 patients without stones, who were prescribed potassium citrate or a liquid diet, and patients with persistent residual urolithiasis, who received potassium citrate or a liquid diet. Stones were classified according to the changes observed during the study, compared with the condition before treatment: stable (no change from the beginning of the trial, with or without residual stone fragments), enlarged (increase in the number and size of remaining calculi or relapse) and decreased (reduction in the number and size or passage of residual stones). The results showed that out of 50 patients who received potassium citrate, the stone formation was not observed in 35 (70%), and the condition remained stable. There was a decrease in ten (20%) and five (10%) cases – an increase in the number and size of stones. Out of 50 patients who had a liquid diet, 19 (38%) remained stable throughout the study, 4 (8%) showed a decrease, and 27 (54%) an increase in the number and size of stones. Out of the total number of patients, 25 (25%) cases of disease relapse were recorded during the entire study, eight were recorded in those who received potassium citrate and 17 in those who did not (Figure 2).

Potassium citrate therapy was statistically significantly effective in patients with residual stones after lithotripsy and relapsing nephrolithiasis [45]. Uric acid calculi account for 10% of all cases of nephrolithiasis and are the second most common types of stones after calcium oxalate and calcium phosphate stones. The most important risk factors for uric acid crystallization and stone formation are low urinary pH (<5.5) and increased uric acid excretion. The main causes of low urine pH are tubular pathology (including gout), chronic diarrhea, and severe dehydration. The development of uric acid nephrolithiasis can be prevented, and uric acid calculi are the few types of urinary tract stones that can be dissolved. Treatment of the disease involves not only hydration (reaching a daily urine volume of more than 2000 ml) but mainly urine alkalinization to a pH level between 6.2 and 6.8. Urine alkalinization using potassium citrate or sodium bicarbonate is a highly effective therapeutic method that helps dissolve existing stones. A low purine diet can reduce uric acid excretion. Potassium citrate is the drug of choice to prevent relapse of urate nephrolithiasis. The use of Allopurinol reduces the incidence of stone formation in patients with hyperuricosuria in the background of relapsing urolithiasis and/or gout [45].

Uric acid stones with or without a calcium component constitute a significant proportion of all types of urinary tract calculi. Understanding the pathophysiology of stone formation is important for determining approaches to treating urolithiasis [46, 47]. The incidence rate of urate nephrolithiasis in different countries ranges from 5 to 40% of all pathology cases [47]. Hyperuricuria, decreased urine volume, and acidity are well-known risk factors for uric acid stones. However, the most important of these is the persistent acidic urine reaction. Gout and myeloproliferative disorders are associated with uric acid nephrolithiasis. Alkalinisation of urine using potassium citrate or sodium bicarbonate are highly effective drugs that help to dissolve existing stones and prevent relapses of nephrolithiasis [48].

### Improving the organization of rehabilitation medical care for patients with urological pathology

To date, the use of potassium citrate has become one of the cornerstones in the treatment of nephrolithiasis. The results of Bulegenova *et al.* [39], Banyra *et al.* [40], Biyashev *et al.* [41] indicated a long-term effect of this drug on the metabolic parameters of urine analysis in patients with urate calculi and a positive effect on the rate of formation. At the Center for the Study of Kidney Stones at the Ducan University Medical Center (USA) during 2000–2006, a retrospective cohort study was conducted among patients who had 24-hour urinalysis results before and after therapy and who continued potassium citrate treatment for at least 6 months (1480 people in total). The average duration of therapy was 41 months (from 6 to 168 months). In general, significant and stable changes in metabolic parameters in the urine were recorded 6 months after the beginning of treatment. These included an increase in urine pH (from 5.90 to 6.46; p<0.0001) and an increase in citrate excretion (from 470 to 700 mg/day; p<0.0001). The rate of stone formation also decreased significantly after administration of potassium citrate – from 1.89 to 0.46 stones per year (p<0.0001). There was a high (68%) degree of remission and a 93% decrease in the rate of stone formation. The administration of potassium citrate leads to significant alkalization of urine and an increase in the citrate level during short-term and long-term therapy, which is manifested in persistent changes in the urinary metabolic profile during 14 years of treatment [42, 43]. In addition, with the long-term use of potassium citrate, the rate of stone formation is significantly reduced, which confirms the effectiveness of the drug in patients with relapsing urolithiasis [44].

Studies in the USA, Canada, Austria, Germany, Great Britain, Poland, Turkey, China, Iran, Korea, Japan, and other countries show a significant prevalence of genitourinary diseases [1–3, 49]. According to the Third National Health and Nutrition Examination Survey in the US, the prevalence of chronic kidney disease in the US adult population was 11%. At the same time, 3.3% had a stage with persistent albuminuria and normal glomerular filtration rate, 3.0% – the disease of stage II, 4.3% – stage III, 0.2% each – stage IV and V [50]. The study found that, apart from arterial hypertension and diabetes, age was a key predictor of chronic kidney disease [1]. Based on the 2007–2009 Healthcare Survey in Canada, the prevalence of kidney disease was 12.5%. Assessment of the prevalence of diseases at stages III-V showed their presence in 3.1% of the population. The prevalence of diabetes mellitus, hypertension, and hypertriglyceridemia was significantly higher in adults with chronic kidney disease than in the population without this pathology [7, 50]. According to epidemiological studies in Germany, 17.4% of patients in healthcare institutions had chronic kidney disease. The prevalence of stages I, II, III, IV–V was 4.6, 4.7, 17.0, and 0.4%, respectively. The prevalence of renal pathology among the population age had a negative upward trend with age and reached maximum values at 70–74 years (23.9%) [8, 51, 52].

Consultative and diagnostic assistance of urological profiles and laboratory and diagnostic examination of patients are essential. Therefore, Abzhaliyeva *et al.* proposed to organize consultative and diagnostic assistance using medical universities [53]. The authors consider using all diagnostic equipment of the consultative and diagnostic center for laboratory and diagnostic examination and the equipment of specialized clinics of higher education institutions as a great advantage, given that almost all consultative and diagnostic center patients need laboratory examinations [54]. A study by Baimbetov *et al.*, focused on improving the organization and treatment of urolithiasis, is devoted to optimizing the regional system of providing urological care to the population [55]. Based on the disadvantages identified in the the regional system of providing healthcare to patients with urolithiasis and taking into account promising approaches, an integral-positive model of organizing medical care for urolithiasis at the regional level was proposed concentrating on this modern surgical urological care in individual high-tech healthcare institutions, providing urgent urological care based on emergency departments of intensive care hospitals, and ambulatory care – based on regional and city consultative and diagnostic centers, timely detection of pathology etc [56–58]. In scientific literature, considerable attention is paid to improving the organization of rehabilitation medical care for patients with urological pathology [59]. In particular, to improve the results of surgical treatment of patients with urolithiasis, it is recommended to carry out rehabilitation measures in a sanatorium immediately after discharge from the hospital for up to 2–3 weeks, develop an individual program of clinical and labor rehabilitation in the immediate postoperative period, depending on the location, size of the stone and the method of surgical treatment of urolithiasis, the implementation of dispensary observation, the improvement of the professional training of urologists in higher education institutions at the undergraduate and postgraduate levels [60, 61].

The assessment of patients' quality of life with genitourinary pathology has become an important area of scientific research. Thus, according to sociological surveys among patients with urolithiasis, 57% of respondents gave an average assessment of the quality of life, only 28% of respondents gave a good rating, and the average score was 3.76. The largest share of respondents who noted poor health was among patients with diseases of the male genital organs (16.4%). For the most part, patients with injuries of the genitourinary system (66.7%), diseases of the male genital organs (66.7%), urolithiasis (65.7%), prostatic hyperplasia (58.3%), and urinary tract infections (55.8%) assessed their condition as mediocre.

## CONCLUSIONS

There is an urgent need to increase the availability and quality of medical services for patients with urological pathology by introducing new technologies and organizational models to achieve universal coverage. However, medical and organizational issues remain unresolved. This requires an integrated approach, taking into account a wide range of pathologies, many influencing factors, challenges, and threats to urological health using modern strategies for the development of urological services, integrative trends, preventive concepts, a patient-oriented approach, public-private partnerships. An analysis of the works of domestic authors and foreign scientific sources made it possible to confirm the scale and social significance of urological health problems, generalize international experience in organizing the provision of urological medical care, and draw a conclusion about the relevance of further research aimed at optimizing the urological service. In addition, there was revealed negative trends towards an increase in the prevalence of diseases of the genitourinary system in many regions, needs to improve monitoring and assessment of epidemiological processes, prevention of urological pathology based on the evidence base, optimisation of medical care organization in the context of healthcare reform.

## ACKNOWLEDGMENTS

### Conflict of interest

There are no conflicts of interest to disclose.

### Authorship

RF took part in project administration, conceptualization, formal analysis, methodology, data curation, writing – review, and editing.
